# Action of low doses of Aspirin in Inflammation and Oxidative Stress induced by aβ_1-42_ on Astrocytes in primary culture

**DOI:** 10.7150/ijms.40959

**Published:** 2020-03-13

**Authors:** Adrian Jorda, Martin Aldasoro, Constanza Aldasoro, Sol Guerra-Ojeda, Antonio Iradi, Jose Mª Vila, Juan Campos-Campos, Soraya L. Valles

**Affiliations:** 1 Department of Physiology, School of Medicine, University of Valencia, Spain.; 2 Faculty of Nursing and Podiatry, University of Valencia, Spain.

**Keywords:** Amyloid-β, aspirin, inflammation, oxidative stress, Alzheimer's disease

## Abstract

Aspirin has been used as anti-inflammatory and anti-aggregate for decades but the precise mechanism(s) of action after the presence of the toxic peptide Aβ_1-42_ in cultured astrocytes remains poorly resolved. Here we use low-doses of aspirin (10^-7^ M) in astrocytes in primary culture in presence or absence of Aβ_1-42_ toxic peptide. We noted an increase of cell viability and proliferation with or without Aβ_1-42_ peptide presence in aspirin treated cells. In addition, a decrease in apoptosis, determined by Caspase 3 activity and the expression of Cyt c and Smac/Diablo, were detected. Also, aspirin diminished necrosis process (LDH levels), pro-inflammatory mediators (IL-β and TNF-α) and NF-ᴋB protein expression, increasing anti-inflammatory PPAR-γ protein expression, preventing Aβ_1-42_ toxic effects. Aspirin inhibited COX-2 and iNOS without changes in COX-1 expression, increasing anti-oxidant protein (Cu/Zn-SOD and Mn-SOD) expression in presence or absence of Aβ_1-42_. Taken together, our results show that aspirin, at low doses increases cell viability by decreasing inflammation and oxidative stress, preventing the deleterious effects of the Aβ_1-42_ peptide on astrocytes in primary culture. The use of low doses of aspirin may be more suitable for Alzheimer's disease.

## Introduction

Alzheimer's disease (AD) causes decline in memory and is the most common neurodegenerative disease implicated in the aging process 1. The prominent features of AD include amyloid plaques, intraneuronal tangles, cell death, inflammatory changes and oxidative stress 2,3,4.

Astroglia are the most prevalent cell type in the brain 5. These cells have roles in the brain protecting against CNS injury and repairing nervous tissue after injury 6. Data from our laboratory demonstrated that astrocytes increase neuronal viability and mitochondrial biogenesis, protecting from oxidative stress and inflammation induced by toxic amyloid peptide 7,8. Also, astrocytes act in neuronal synapses, regulate the blood-brain barrier, providing nutrients to the nervous system and maintaining ion and metabolite balance, and also propagate calcium currents, release gliotransmitters, growth factors and inflammatory mediators 9,10,11. Astrogliosis is produced by a reaction from astrocytes to inflammation, oxidative stress and cell death producing toxic products and inflammatory agents and oxidative stress mediators 3,12. In astrocytes, complex changes and specific conflicts occur in different brain regions during the development of AD. The number of reactive astrocytes increases in AD, phagocytizing and reducing amyloid β (Aβ) deposition because these cells surround amyloid plaques and secrete proinflammatory factors 13,14.

Acetylsalicylic acid (aspirin) is used frequently as a member of the nonsteroidal anti-inflammatory drugs (NSAIDs) group 15. Aspirin induces its effects by inhibiting cyclooxygenase (COX) and suppresses prostaglandins 16,17. Two main isoforms of COX exist, COX-1 and COX-2. COX-1 is involved in the synthesis of thromboxane A_2_ (TXA_2_) 18 and COX-2 in prostacyclin biosynthesis 19,20. In epidemiological studies of AD, a high dose of aspirin produces lower prevalence of AD 21 (Nilsson *et al*. 2003). Other authors using low-dose aspirin treatment indicated promising results for aspirin 22. In this study, we were interested in exploring the action of aspirin in both inflammatory and ROS (reactive oxygen species) events associated with Alzheimer's disease (AD) by using the amyloid β_1-42_ in astrocytes in primary culture. The accumulation and precipitation of Aβ_1-42_ peptide has a neuropathological role associated with AD. However the Aβ_40-1_ peptide has non-toxic effects and is used as control.

## Material and Methods

### Materials

This study was approved by the Bioethics Committee of the School of Medicine of the University of Valencia, and the local Government of Valencia, Spain (2016/VSC/PEA/00220). All animals (Wilson rats from Charles River laboratory) were handled according to the recommendations of the Committee and with access to food and water. Animals were sacrificed with pentobarbital by the veterinary personnel. No randomization was performed to allocate subjects in the study. Rats (6 months old) were housed alone for 21 days during pregnancy. Female rat fetuses were obtained at day 21 of pregnancy prior to delivery. The fetal cortex was obtained to create a culture of astrocytes. Every assay was performed 3, 4 or 5 times, from different mother rats. No blinding procedures for each culture were made. Female rats were excluded if they were pregnant before the study. Rats were also excluded if the delivery of rats was 21 days before pregnancy.

Dulbecco's modified Eagle's medium (DMEM) and fetal bovine serum (FBS) were obtained from Gibco (Gibco Invitrogen Corporation, Barcelona, Spain). The oligomers Aβ (40-1 and 1-42), were prepared following manufacturer's instructions (Sigma-Aldrich biotechnology). Briefly, the peptides were dissolved in 100 µM phosphate buffered saline (PBS) and preparations were heated for 24 h at 37°C for assembly of the oligomers. Aspirin was obtained from Sigma-Aldrich biotechnology and dissolved in Krebs solution to the proper final concentration (10^-7^ M). 3-(4,5-dimethyl-2-thiazolyl)-2,5-dipheniyl-2H-tetrazolium bromide (MTT) was purchased from Sigma Chemical Co. (St Louis, MO). Enzyme-linked immunosorbent assay (ELISA) kits for IL-1β (Interleukin 1-β) and TNF-α (Tumor necrosis-α) from Pierce Biotechnology, Inc. (Rockford, USA). Western Blot Chemiluminescent Detection System (ECL) was from Amersham (Amersham Biosciences, Barcelona, Spain). Monoclonal anti-cytochrome C (Cyt c) antibody (1:500), monoclonal anti-Smac/Diablo antibody (Smac/Diablo) (1:500), monoclonal anti-nuclear factor κB antibody (NF-κB) (1:1000), monoclonal anti-Mn superoxide dismutase antibody (Mn-SOD) (1:500), monoclonal anti-cyclooxygenase 1 antibody (COX-1) (1:500), monoclonal anti-cyclooxygenase 2 antibody (COX-2) (1:500), monoclonal anti-inducible nitric oxide synthase antibody (iNOS) (1:500) from Santa Cruz Biotechnology (Madrid, Spain). Monoclonal anti-peroxisome proliferator-activated receptor antibody (PPAR-γ) (1:500) from Sigma Aldrich (Madrid, Spain). Polyclonal anti-Cu/Zn superoxide dismutase antibody (Cu/Zn-SOD) (1:500) from Assay Designs (Madrid, Spain). Monoclonal anti-tubulin antibody (1:1000) from Cell Signaling (Beverly, MA, USA). All other reagents were analytical or culture grade purity.

## Methods

### Primary Culture of Cortical Astrocytes

Cerebral cortical astrocytes were isolated from rat fetuses of 21 days gestation. Fetuses were obtained by caesarean section and decapitated. Cerebral cortices were removed and triturated 10-15 times through a Pasteur pipette. The cell suspension was filtered through nylon mesh with a pore size of 90 μm and diluted in DMEM containing 20% fetal bovine serum (FBS) supplemented with L-glutamine (1%), HEPES (10 mM), fungizone (1%), and antibiotics (1%). Cells were plated on T75 culture flask pretreated with poli-L-lysine. Cultures were maintained in a humidified atmosphere of 5% CO2/95% air at 37°C during 20 days. After 1 week of culture, the FBS content was reduced to 10%, and the medium was changed twice a week. By immunocytochemistry, 97% of cells are GFAP positive (data not shown).

Four groups were used. Group A, control received Aβ_40-1_ peptide, Group B, Aβ_40-1_ peptide + aspirin, Group C, Aβ_1-42_ toxic peptide and Group D, Aβ_1-42_ toxic peptide + aspirin. Initially, we used Aβ_42-1_ but due to its high cost, we assayed Aβ_40-1_ as we have used before 7.

### MTT assay

Cell viability of the cultures was determined by the MTT assay. Cells were plated in 96 well culture plate and incubated with Asp during 24 h at 10^-11^ M, 10^-9^ M, 10^-7^ M, 10^-5^ M, Aβ_1-42_ 15 µM or with Aβ_1-42_ 15 µM + 10^-7^ M Asp. After cell treatments, the medium was removed and the cortical cells were incubated with red free medium and MTT solution [0.5 mg/ml, prepared in phosphate buffer saline (PBS) solution] for 4 h at 37ºC. Finally the medium was removed and formazan particles were dissolved in dimethyl sulfoxide (DMSO). Cell viability, defined as the relative amount of MTT reduction, was determined by spectrophotometry at 570 nm.

### Trypan Blue Assay

Trypan blue exclusion assay was used to count the living cells and monitor cell proliferation. Astrocytes were isolated and seeded at 7x10^4^ cells/35 mm dish. After 5 days of culture, cells were incubated without (control, C), with Asp (10^-7^ M), Aβ_1-42_ 15 µM or with Aβ_1-42_ 15 µM + 10^-7^ M Asp for 24 h. 1.5% trypan blue solution was applied to astrocytes cultures at room temperature for 3 min.

### Lactate Dehydrogenase (LDH) Assay

To evaluate plasma membrane integrity, LDH release was determined by monitoring the leakage of the cytosolic LDH to the extracellular medium. LDH was measured spectrophotometrically at 340 nm, following the rate of conversion of reduced nicotinamide adenine dinucleotide to oxidized nicotinamide adenine dinucleotide.

### Caspase 3 Activity Assay

Caspase 3 activity was measured in cytosolic fractions by using a highly sensitive colorimetric substrate, N-acetyl-Asp-Glu-Val-Asp p-nitroanilide (Ac-DEVD-pNA) following manufacturer´s instructions (CalBiochem, La Jolla, CA). Enzyme activity was calculated using manufacturer´s formulae, as pmol/min.

### Cytokine Determination, IL-1β and TNFα

Cells were seeded, and at time of assay, the red phenol medium was removed and replaced by PBS containing 1 mg/ml bovine serum albumin (BSA), either in the presence or absence of Asp (10^-7^ M), with Aβ_1-42_ 15 µM or Aβ_1-42_ 15 µM + 10^-7^ M Asp). IL-1β and TNF-α concentration (pg/ml) were ascertained using ELISA kits (Pierce Biotechnology, Inc.).

### Western Blot Analysis

Cultured cells were treated with lysis buffer and then mechanically degraded to release the proteins. Protein concentration was determined using modified Lowry method. Loading buffer (0.125 M Tris-HCl, pH 6.8, 2% SDS, 0.5% (v/v) 2-mercaptoethanol, 1% bromophenolblue and 19% glycerol) was added to protein sample and heated for 5 min at 95ºC. Proteins (20 µg) were separated on SDS-PAGE gels and transferred to nitrocellulose membranes in a humid environment using a transfer buffer (25 mM Tris, 190 mM glycine, and 20% methanol). Membranes were blocked with 5% milk in TBS-T (0.05% Tween-20) and incubated with primary antibodies overnight at 4ºC. Membranes were washed 3 times with wash buffer TBS-T (TBS, 0.2% Tween-20) and incubated with a secondary anti-rabbit IgG or anti-mouse IgG antibody conjugated to the enzyme horseradish peroxidase (HRP) for 1 h. Membranes were washed three times and proteins were detected using the ECL method as specified by the manufacturer. Autoradiography signals were assessed using digital image system ImageQuant LAS 4000 (GE Healthcare). Densitometry is the quantitative measurement of optical density in a photographic paper or photographic film, due to exposure to light. Concentration of protein was determined by densitometry analysis, expressed as arbitrary units relative to tubulin.

### Statistical Analyses

All values are expressed as mean ± S.D. The differences between groups were determined with unpaired Student´s t-test. All statistical analyses were performed using the GraphPad Prism software (GrapshPad Software Inc., San Diego, CA, USA). Statistical significance was accepted at *p* ≤ 0.05.

## Results

### Asp and Cell Viability

**Figure 1:**
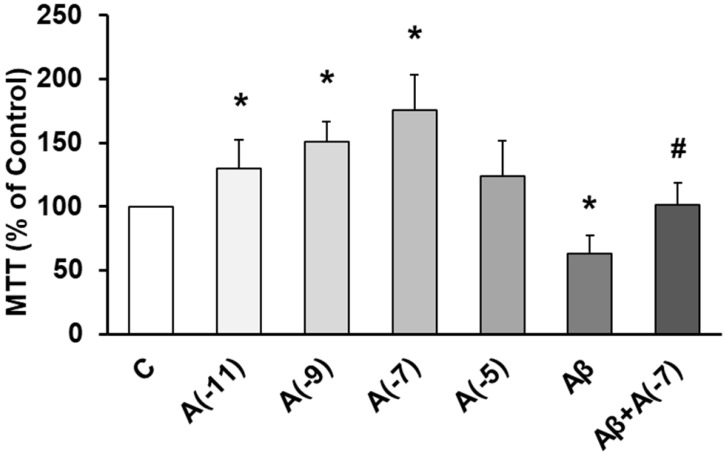
** Cell viability was determined by MTT assay in cells treated during 24 h.** Astrocytes were incubated without Asp (control, C), with Asp at different concentrations (10^-11^, 10^-9^, 10^-7^ and 10^-5^ M), with Aβ_1-42_ (15 µM) or Aβ_1-42_ (15 µM) + Asp (10^-7^ M) for 24 h. Data are means ± SD of four independent experiments (three different rats). *p < 0.05 *vs*. control. # *p* < 0.05 *vs* Aβ_1-42_ treated cells.

The role of Asp on cell viability was studied using MTT conversion assay. Fig. 1 shows that incubation with Asp at 10^-11^ M, 10^-9^ M, and 10^-7^ M significantly increased astrocyte viability *vs* control. On the other hand, Aβ_1-42_ significantly decreased cell viability (30%) compared to control cells. After incubation with Aβ_1-42_ + 10^-7^ M Asp, no significant changes were detected compared to control astrocytes and contrarily, an increase in cell viability was detected compared to cells with Aβ_1-42_ peptide alone.

Trypan blue exclusion assay was used to count the living cells and monitor cell proliferation. Astrocytes were isolated and seeded at 7x10^4^ cells/35 mm dish. After 5 days of culture, cells were incubated without (control, C) or with Asp 10^-7^ M, Aβ_1-42_ 15 µM or Aβ_1-42_ 15 µM + Asp 10^-7^ M for 24 h. In control conditions proliferation was 0.93%, and previous incubation with Asp (10^-7^ M) increased proliferation by 9.53%. On the other hand, in presence of Aβ_1-42_ proliferation decreased 12.96% and with Aβ_1-42_ + Asp 10^-7^ M only decreased 5.37% (Table 1).

### LDH and Caspase 3

**Figure 2:**
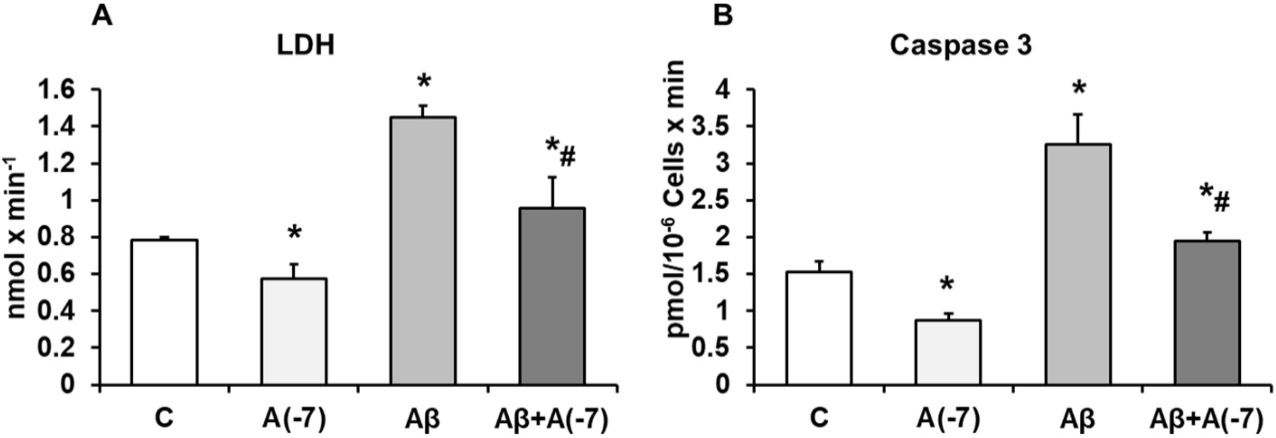
** Lactate dehydrogenase and caspase 3 activity.** Astrocytes were incubated without Asp (control, C), with Asp (10^-7^ M), Aβ_1-42_ (15 µM) or Aβ_1-42_ (15 µM) + Asp (10^-7^ M) for 24 h. Panel A: Lactate dehydrogenase from supernatants of astrocytes. Panel B: Caspase 3 activity. Data are means ± SD of four independent experiments (four different rats). *p < 0.05 *vs*. control cells. #*p* < 0.05 *vs*. Aβ_1-42_ treated cells.

Incubation of the astrocytes with Asp 10^-7^ M for 24 h decreased significantly LDH values (21%) compared with control cells. With Aβ_1-42_ (15 µM) an increase of LDH release (55%) was detected compared with control cells and this data was reversed with Asp (10^-7^ M) to control values (Fig. 2A).

Incubation with Asp 10^-7^ M for 24 h, decreased Caspase 3 activity significantly (45%) compared to control cells, whereas Aβ_1-42_ (15 µM) activity was increased in presence of Asp (10^-7^ M), and prevented the toxic peptide effect (Fig 2B), indicating reduction of apoptosis when Asp is present on the culture with Aβ_1-42_.

### Cytochrome c and Smac/DIABLO Expression

**Figure 3:**
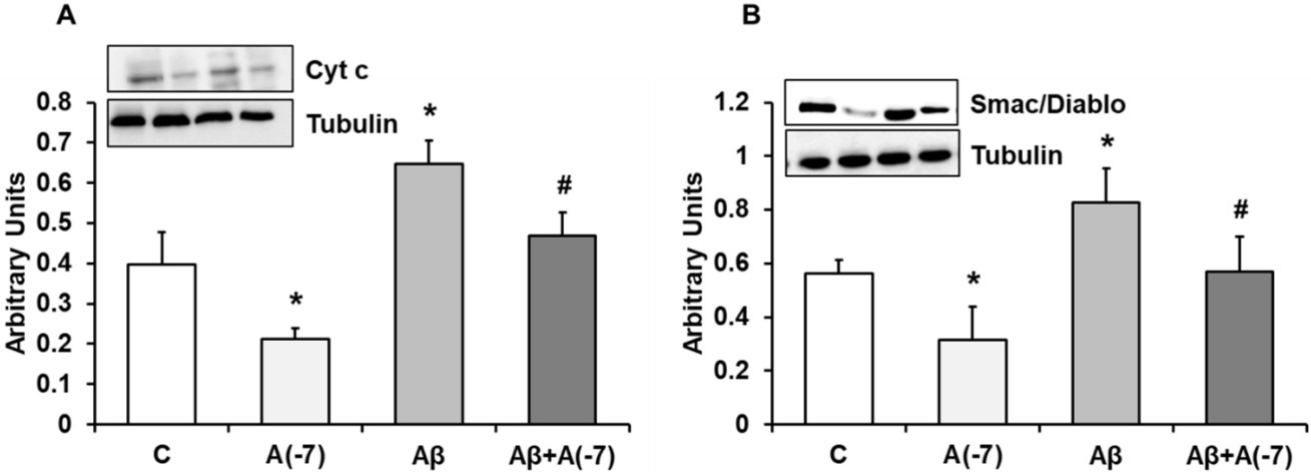
** Cytocrhome c and Smac/Diablo expression.** Astrocytes were incubated without Asp (control, C), with Asp (10^-7^ M), Aβ_1-42_ (15 µM) or Aβ_1-42_ (15 µM) + Asp (10^-7^ M) for 24 h and collected to determine Cytocrhome c (panel A) or Smac/Diablo (panel B) protein expression by Western-blot. A representative immunoblot is shown in the top panel. Data are means ± SD of four independent experiments (four different rats). *p < 0.05 *vs*. control cells. #*p* < 0.05 *vs*. Aβ_1-42_ treated cells.

Fig. 3A shows cytochrome c expression in astrocytes in primary culture. Asp decreased cytochrome c expression by 2.2-fold at 10^-7^ M. Aβ_1-42_ increased cytochrome c expression compared to control cells. On the contrary, Asp (10^-7^ M) significantly reversed the cytochrome c increase expression induced by Aβ_1-42_. Fig. 3B shows Smac/Diablo expression in astrocytes in primary culture. Asp significantly decreased Smac/Diablo protein expression compared to control cells. Addition of Aβ_1-42_ significantly increased Smac/Diablo expression (1.3-fold) compared with control cells. Furthermore, Asp 10^-7^ M decreased Smac/Diablo expression induced by Aβ_1-42_ to control values. Consequently Asp addition produced a protective effect against the amyloid toxic peptide (Fig. 3).

### IL-1β and TNF-α Pro-inflammatory Cytokines

**Figure 4:**
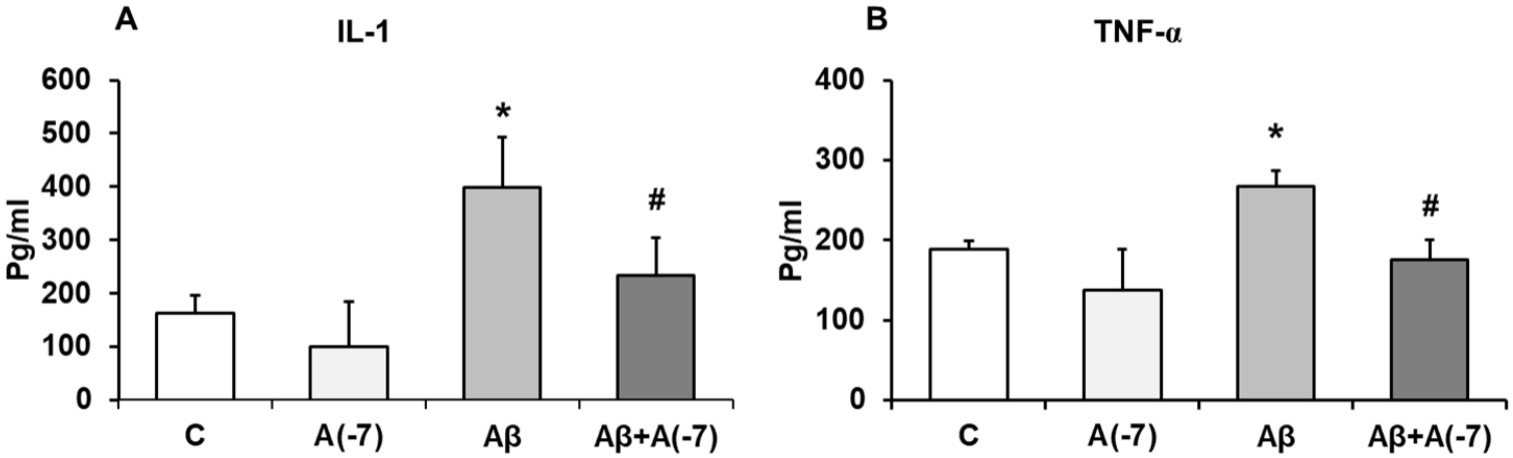
** IL-1β and TNF-α determination.** Astrocytes were incubated without Asp (control, C), with Asp (10^-7^ M), Aβ_1-42_ (15 µM) or Aβ_1-42_ (15 µM) + Asp (10^-7^ M). Cell culture supernatants were harvested and IL-1β (Panel A) and TNF-α (Panel B) were determined by ELISA. Values are means ± SD from four independent experiments (four different rats). *p < 0.05 *vs* control. #*p* < 0.05 *vs*. Aβ treated cells.

Secretion of the pro-inflammatory mediators, IL-1β and TNF-α, were detected by ELISA. Fig 4 shows that, in astrocytes, Asp (10^-7^ M) decreased 1.6-fold IL-1β release compared with control values (Fig 4A). Conversely, Aβ_1-42_ addition increased 2.5-fold IL-1β secretion compared with control cells. Aβ_1-42_ + Asp 10^-7^ M decreased IL-1β liberation to control values.

Fig. 4B shows that Asp (10^-7^ M) produced a significant decrease of TNF-α secretion (3.1-fold) compared to control cells. Aβ_1-42_ addition increased TNF-α liberation 1.5-fold. Moreover, Asp (10^-7^ M) prevented this toxic effect produced by Aβ_1-42_ peptide.

### NF-κB and PPAR-γ Expression

**Figure 5:**
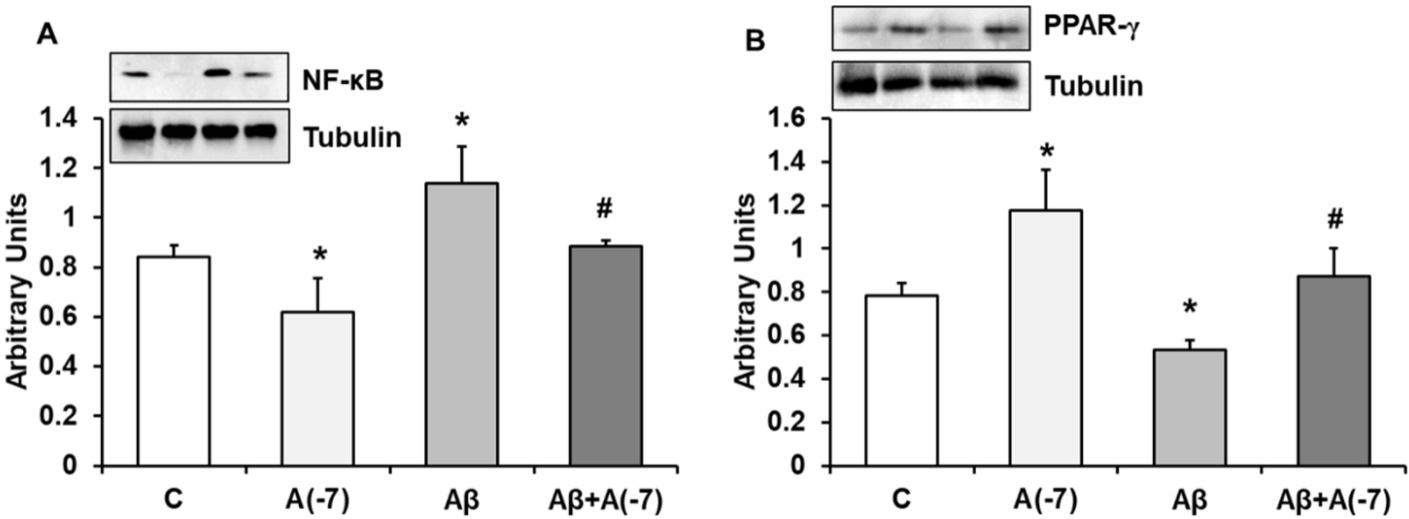
** NF-ᴋB and PPAR-γ expression.** Astrocytes were incubated without Asp (control, C), with Asp (10^-7^ M), Aβ_1-42_ (15 µM) or Aβ_1-42_ (15 µM) + Asp (10^-7^ M) for 24 h and collected to determine NF-ᴋB (panel A) or PPAR-γ (panel B) protein expression by Western-blot. A representative immunoblot is shown in the top panel. Data are means ± SD of four independent experiments (four different rats). *p < 0.05 *vs*. control cells. #*p* < 0.05 *vs*. Aβ_1-42_ treated cells.

NF-κB is a transcription factor that regulates positively gene expression of pro-inflammatory proteins. Fig. 5A shows that Asp decreased this protein expression about 1.25-fold compared to control astrocytes. When Aβ_1-42_ was added an increase (1.5-fold) of this transcription factor was detected compared to control results. Asp (10^-7^ M) addition returned NF-κB to control values.

PPARs family regulates negatively gene expression of pro-inflammatory proteins. Fig. 5B shows PPAR-γ expression in astrocytes in culture. Asp increased PPAR-γ expression 1.5-fold at 10^-7^ M. Addition of Aβ_1-42_ decreased 1.6-fold this transcription factor compared to control results. Asp (10^-7^ M) addition returned PPAR-γ expression to control values.

### COX-2 and iNOS Expression

**Figure 6:**
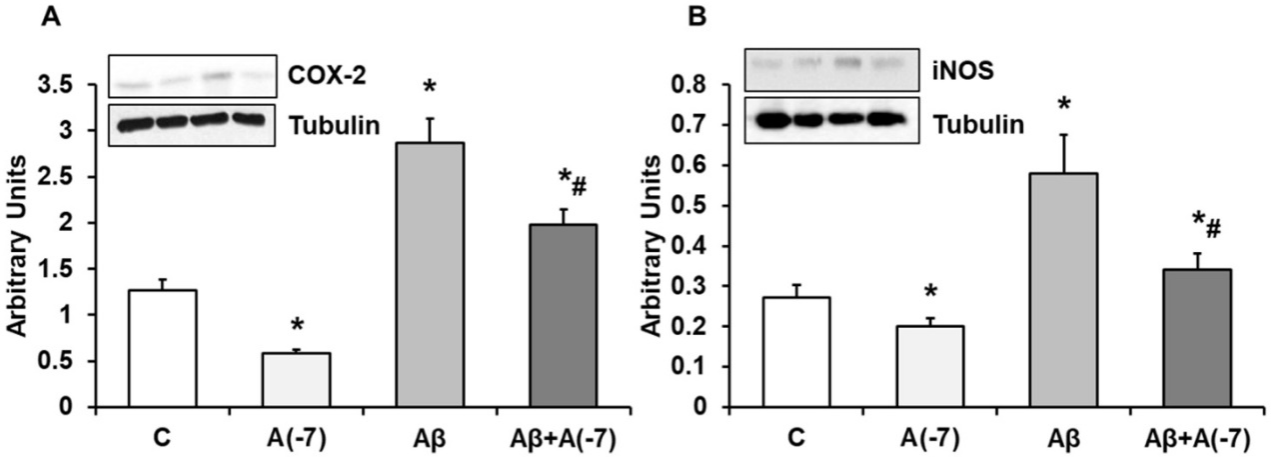
** COX-2 and iNOS expression.** Astrocytes were incubated without Asp (control, C), with Asp (10^-7^ M), Aβ_1-42_ (15 µM) or Aβ_1-42_ (15 µM) + Asp (10^-7^ M) for 24 h and collected to determine COX-2 (panel A) or iNOS (panel B) protein expression by Western-blot. A representative immunoblot is shown in the top panel. Data are means ± SD of four independent experiments (four different rats). *p < 0.05 *vs*. control cells. #*p* < 0.05 *vs*. Aβ_1-42_ treated cells.

In Fig. 6, we detected a reduction of COX-2 (panel A) expression after addition of Asp (10^-7^ M) compared with control values. The presence of the toxic peptide Aβ_1-42_ increased the expression of COX-2 that was reversed by the incubation with Asp 10^-7^ M to control values. On the contrary, no changes were detected in COX-1 expression at any experimental condition (Data not shown). Fig. 6 (panel B) demonstrates a significand decrease in iNOS protein expression after Asp 10^-7^ M addition compared to control. Aβ_1-42_ produced a significant increase of iNOS expression. On the other hand, the presence of both the toxic peptide and Asp 10^-7^ M reduced significantly iNOS expression compared to Aβ_1-42_.

### Expression of CU/ZN-SOD and Mn-SOD Proteins

**Figure 7:**
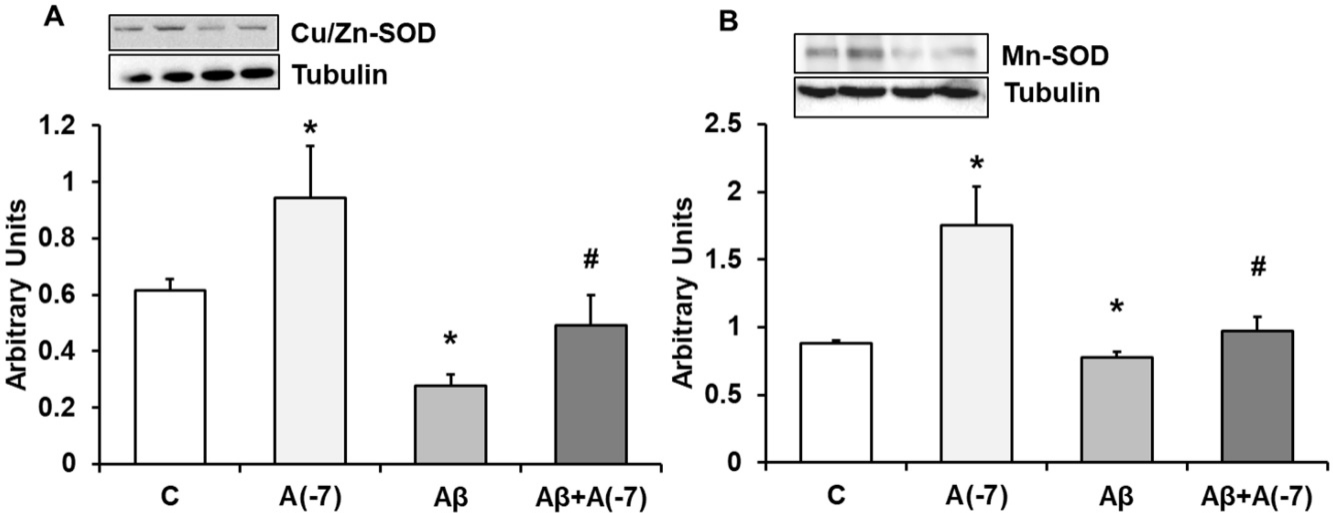
** Cu/Zn-SOD and Mn-SOD expression.** Astrocytes were incubated without Asp (control, C), with Asp (10^-7^ M), Aβ_1-42_ (15 µM) or Aβ_1-42_ (15 µM) + Asp (10^-7^ M) for 24 h and collected to determine Cu/Zn-SOD (panel A) or Mn-SOD (panel B) protein expression by Western-blot. A representative immunoblot is shown in the top panel. Data are means ± SD of four independent experiments (four different rats). *p < 0.05 *vs*. control cells. #*p* < 0.05 *vs*. Aβ_1-42_ treated cells.

In astrocytes, Asp 10^-7^ M increased Cu/Zn-SOD (panel A) and Mn-SOD (panel B) expression compared to control cells (Fig. 7). In the presence of the Aβ_1-42_ toxic peptide, a decrease of both proteins compared with control values was reversed by Asp.

## Discussion

In this study we show that aspirin, at low-doses, protects from Aβ_1-42_ toxic peptide actions in astrocytes in primary culture, indicant the convenience to use low doses to obtain better benefices of aspirin. The aspirin increases cell viability and proliferation, decreases apoptosis (Caspase 3, Cyt c and Smac/Diablo) and necrosis (LDH), in the presence or absence of Aβ_1-42_ peptide. Moreover, the aspirin decreases pro-inflammatory mediators (IL-β and TNF-α) and NF-κB expression and increases anti-inflammatory PPAR-γ protein after addition of Aβ_1-42_. As also inhibits COX-2 and iNOS without changes in COX-1 expression and increases anti-oxidant proteins (Cu/Zn-SOD and Mn-SOD) expression in the presence or absence of Aβ_1-42_.

The role of astrocytes in the brain has been reviewed 23,24,25. It is reported that astrocytes protect neurons against Aβ-amyloid peptide, decreasing inflammation, and oxidative stress, and increasing cell viability and mitochondrial biogenesis 7,8,26.

Low-dose of aspirin has been reported to reduce the incidence of Alzheimer's disease 27 and also the donation of its acetyl group to molecules that suppress protein aggregation in neurodegenerative diseases, including AD 28. This mechanism could be implicated in the astrocytic protection observed in our study. Aβ_1-42_ peptide induces reduction in cell viability and increases apoptosis by cytochrome c and Smac/Diablo pathways 7,29. However, simultaneous treatment with aspirin of Aβ_1-42_ treated cells protects them from neurotoxicity 29. Reactive gliosis increases glial fibrillary acidic protein (GFAP) in AD where it is highly expressed and remarkably, aspirin produces a reduction in GFAP synthesis by blocking NF-κB in astrocytes culture 30. It is reported that high doses aspirin can help reduce chronic AD inflammation 30. However, our results show that aspirin, at low concentration (10^-7^ M), increases cell viability, diminishing necrosis and apoptosis. Burke *et al*. (2006) 31 reported that human daily ingestion of aspirin, 80 to 350 mg, produces plasma salicylate levels of 2.4 to 9.7 µg/ml, corresponding to 1.3 × 10^-6^ to 5.4 x 10^-5^ M of aspirin in the culture medium 31. According to these results, the concentrations used in our experiments correspond to doses lower than 70 mg/day, considered as low doses of aspirin 32. However, aspirin increases apoptosis in gastric mucosal cell line in a caspase-dependent manner through Smac/Diablo pathway 33. Our results differ due to aspirin concentration used. Redlak *et al*. (2005) 33 used a concentration of 4 × 10^-2^ M whereas in our study we used 10^-7^ M.

Our results indicate that low concentrations of aspirin may decrease apoptosis as opposed to high doses, protecting without increasing the adverse mechanisms observed in other paper.

After Aβ_1-42_ addition, we detected an increase in COX-2 without changes in COX-1 expression. Both COX proteins could lead to adverse cellular effects derived from TXA_2_ and prostacyclin biosynthesis. Using LPS (bacterial lipopolysaccharide) to determine COX isoform expression in astrocytes, Font-Nieves *et al*. (2012) 34 showed a strong induction of COX-2 through an NF-κB-dependent mechanism 34. In chronic inflammation and in the progression of neurodegenerative diseases, NF-κB activation plays an important role in astrocytes in primary culture 35, according to previous data obtained in our laboratory indicating that Aβ addition is also associated with an increase in NF-κB activity in astrocytes 7. Lee et al. (2018) 36, have indicated that downregulation of inflammatory molecules such as iNOS and different cytokines (TNF-α, IL-1β and IL-6) could be a consequence of the NF-κB and MAP kinases inhibition in astrocytes 36. Furthermore, NF-κB binding sites are present in the promoters of iNOS and COX-2 and are essential to modulate their transcription 37,38. Results of Yao *et al*. (2014) 39 demonstrated that aspirin normalizes COX-2 and iNOS over-expression in astrocytes in part through inhibition of the NF-κB pathway 39. As these authors indicated, we show a reduction in the expression of iNOS, COX-2 and NF-κB in the presence of aspirin.

Aspirin has additional targets in humans, besides the cyclooxygenases. Salicylic acid (SA), the primary metabolite of aspirin, binds human glyceraldehyde 3-phosphate dehydrogenase (GAPDH) and suppresses nuclear translocation and cell death 40. It is known that GAPDH plays an important role in neurodegenerative diseases, including AD 41.

In neurodegenerative diseases such as AD, treatment with non-steroidal anti-inflammatory drugs (NSAIDs) has been reported 42 and COX-1 and COX-2 are the targets of those drugs. Furthermore both COX isoforms regulate PPARγ activity through prostaglandin synthesis 43. Also, PPARγ has been shown to inhibit the expression of proinflammatory genes, such as iNOS 44,35 and has several inhibitory effects on inflammation, including reduction of NF-κB transcriptional activities and promotes anti-inflammatory mediators 43. Potent synthetic antidiabetics such as rosiglitazone are agonists for PPAR-γ 45,46 and many NSAIDs are also PPAR-γ agonists 47. Furthermore, reduction of Aβ induced by indomethacin or naproxen by inhibiting BACE1 (beta-site APP-cleaving enzyme 1) activity occurs in a PPAR-γ dependent manner 48. In our study, we detected a decrease of PPAR-γ expression after Aβ addition that was reversed by treatment with aspirin. It is possible that aspirin could inhibit Aβ synthesis through the increment of PPAR-γ.

In this study we also investigated the possible antioxidant properties of aspirin using Aβ_1-42_ toxic peptide as a model. We showed an increase expression of Mn-SOD and Cu/Zn-SOD proteins after aspirin addition in culture of astrocytes with and without the toxic peptide. In agreement with our results, Dairam *et al*. (2006) 49 have demonstrated the antioxidant and neuroprotective effects of NSADs in an AD rat model 49. Furthermore, aspirin-elicited lipoxin A_4_ inhibited ROS synthesis induced by LPS in microglial cells 50. Also, aspirin minimizes the effect of free radicals induced by LPS in rat dopaminergic neurons 51. Liu *et al*. (2017) 52 indicated that aspirin at doses of 75 and 100 mg/day, similar to that used in our study, stimulates the levels of superoxide dismutase (SOD). On the other hand, production of L-NMMA in microcirculation is inhibited by aspirin, due to cyclooxygenase inhibition or SOD increase 53,54.

Also, aspirin exerts pro-oxidant effects on Mn-SOD-deficient yeast cells causing apoptosis with mitochondrial involvement 55. Furthermore, aspirin on focal cerebral ischemia-reperfusion rats reduced MDA content 56.

## Conclusion

**Figure 8:**
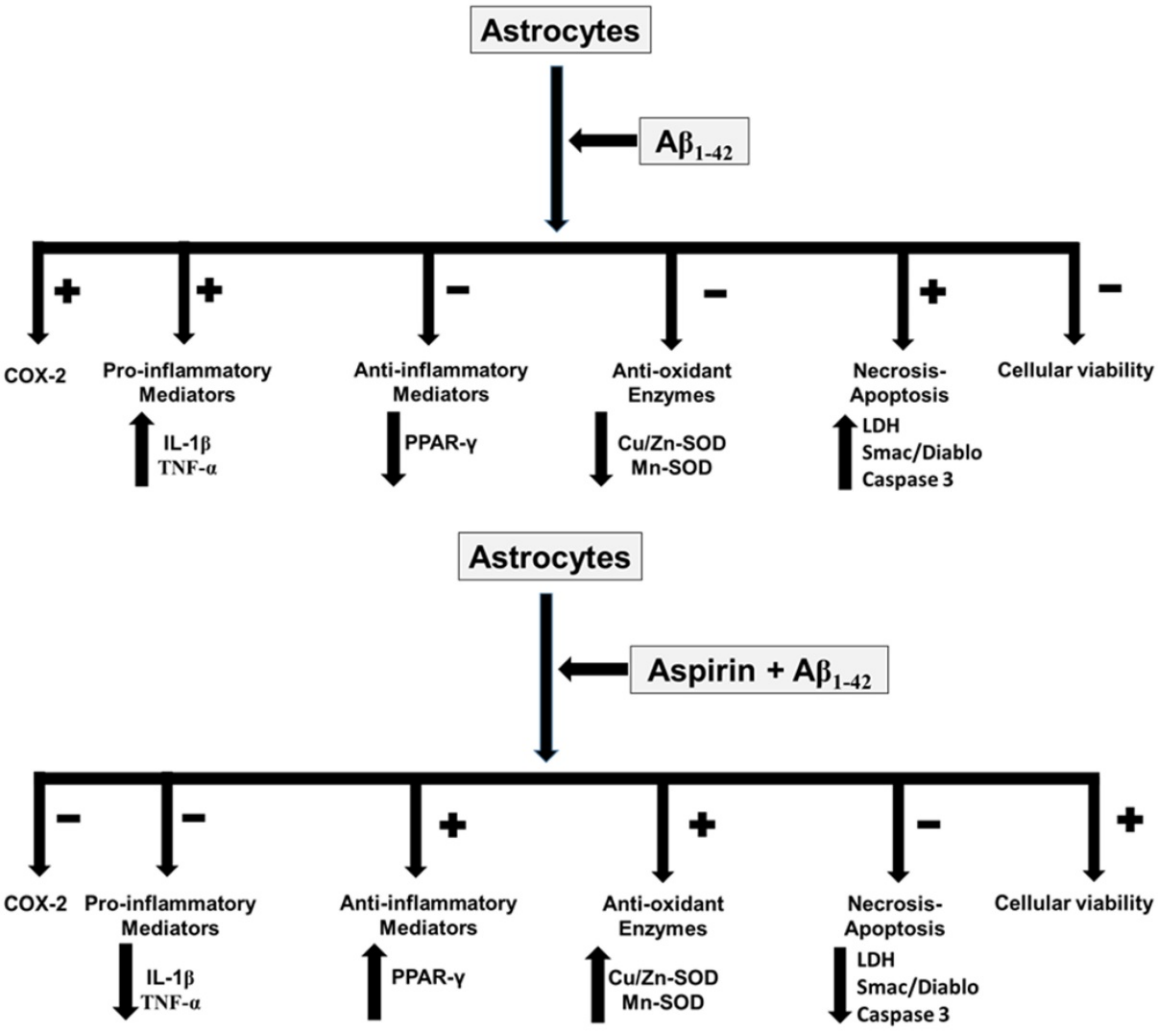
** Aspirin effects in astrocytes in primary culture**. Asp increases cell viability, anti-inflammatory response and anti-oxidant proteins. On the other hand, Asp decreases pro-inflammatory mediators, necrosis and apoptosis.

In conclusion, our results indicate that aspirin, at low doses, prevents toxic effects induced by Aβ_1-42_ peptide in astrocytes in primary culture, increasing cell viability and proliferation while decreasing apoptosis and necrosis. The key finding of our study is that aspirin at low doses prevents oxidative stress and inflammation induced by Aβ_1-42_ toxic peptide. So, the administration of low doses of aspirin could be useful in AD patients (Fig. 8).

## Figures and Tables

**Table 1:** Astrocytes proliferation and counting living cells

	Seeding cells	5 days of culture	After 24 h of incubation	% Proliferation
C	7	12.72±0.19	12.83±0.20	+0.93
Asp (10-7 M)	7	12.70±0.11	13.91±0.16*	+9.53
Aβ	7	12.65±0.23	11.01±0.32*	-12.96
Aβ + Asp (10-7 M)	7	12.75±0.24	12.10±0.14*	-5.37

Astrocytes were isolated and seeded at 7x10^4^ cells/35 mm dish during 5 days. At this time, cells were incubated without Asp (control, C), with Asp (10^-7^ M), Amyloid β_1-42_ (15 µM) or Amyloid β_1-42_ (15 µM) + Asp (10^-7^ M) for 24 h. Trypan blue exclusion was used to count the living cells and monitor cell proliferation. Data are mean ± SD of five independent experiments (four different rats). *p < 0.05 *vs.* control.

## Abbreviations

MTT: 3-(4,5-dimethyl-2-thiazolyl)-2,5-dipheniyl-2H-tetrazolium bromide
Asp: Acetylsalicylic acid: aspirin
AD: Alzheimer's disease
Aβ: Amyloid β
Cu/Zn: superoxide dismutase
COX: Cyclooxygenase
Cyt c: Cytochrome c
GFAP: Glial fibrillary protein
NF-κB: Nuclear factor κB
PPAR-γ: Peroxisome proliferator-activated receptor
